# The Genomics and Population Genomics of the Light Brown Apple Moth, *Epiphyas postvittana*, an Invasive Tortricid Pest of Horticulture

**DOI:** 10.3390/insects13030264

**Published:** 2022-03-07

**Authors:** Amali H. Thrimawithana, Chen Wu, John T. Christeller, Robert M. Simpson, Elena Hilario, Leah K. Tooman, Doreen Begum, Melissa D. Jordan, Ross Crowhurst, Richard D. Newcomb, Alessandro Grapputo

**Affiliations:** 1 The New Zealand Institute of Plant and Food Research Limited, Auckland 1025, New Zealand; amali.thrimawithana@plantandfood.co.nz (A.H.T.); chen.wu@plantandfood.co.nz (C.W.); elena.hilario@plantandfood.co.nz (E.H.); ltooman@gmail.com (L.K.T.); doreenb@douglas.co.nz (D.B.); melissa.jordan@plantandfood.co.nz (M.D.J.); ross.crowhurst@plantandfood.co.nz (R.C.); 2 The New Zealand Institute of Plant and Food Research Limited, Palmerston North 4410, New Zealand; john.christeller@gmail.com (J.T.C.); robert.simpson@plantandfood.co.nz (R.M.S.); 3 School of Biological Sciences, University of Auckland, Auckland 1010, New Zealand; 4 Dipartimento di Biologia, Università degli Studi di Padova, 35131 Padova, Italy; alessandro.grapputo@unipd.it

**Keywords:** Lepidoptera genome, pest species, invasive species, *Epiphyas postvittana*, light brown apple moth, polyphagy, population genomics

## Abstract

**Simple Summary:**

In this study, we produced a genomic resource for the light brown apple moth, *Epiphyas postvittana*, to understand the biological basis of adaptation to a high number of hosts (polyphagy) and the invasive nature of this and other lepidopteran pests. The light brown apple moth is an invasive pest of horticultural plants, with over 500 recorded plant hosts. With origins in Australia, the pest has subsequently spread to New Zealand, Hawaii, California and Europe, causing significant economic losses for fruit producers. Comparative genomic analyses with other lepidopteran genomes indicate that a high proportion of the genome is made up of repetitive sequences, with the majority of the known elements being DNA transposable elements and retrotransposons. Twenty gene families show significant expansions, including some likely to have a role in its pest status. Finally, population genomics, investigated by a RAD-tag approach, indicated likely patterns of invasion and admixture, with Californian moths most probably being derived from Australia.

**Abstract:**

The light brown apple moth, *Epiphyas postvittana* is an invasive, polyphagous pest of horticultural systems around the world. With origins in Australia, the pest has subsequently spread to New Zealand, Hawaii, California and Europe, where it has been found on over 500 plants, including many horticultural crops. We have produced a genomic resource, to understand the biological basis of the polyphagous and invasive nature of this and other lepidopteran pests. The assembled genome sequence encompassed 598 Mb and has an N50 of 301.17 kb, with a BUSCO completion rate of 97.9%. *Epiphyas postvittana* has 34% of its assembled genome represented as repetitive sequences, with the majority of the known elements made up of longer DNA transposable elements (14.07 Mb) and retrotransposons (LINE 17.83 Mb). Of the 31,389 predicted genes, 28,714 (91.5%) were assigned to 11,438 orthogroups across the Lepidoptera, of which 945 were specific to *E. postvittana*. Twenty gene families showed significant expansions in *E. postvittana*, including some likely to have a role in its pest status, such as cytochrome p450s, glutathione-S-transferases and UDP-glucuronosyltransferases. Finally, using a RAD-tag approach, we investigated the population genomics of this pest, looking at its likely patterns of invasion.

## 1. Introduction

The light brown apple moth, *Epiphyas postvittana*, is a polyphagous, invasive tortricid moth [1]. Larvae have been recorded from more than 500 host plant species in 363 genera, from 121 families across vascular plants [2]. The ability to tolerate the toxicity associated with many of the chemical components of leaves from different plants has been attributed to the diversity of detoxification enzymes expressed in the larval gut [3,4]. In Australia and New Zealand, *E. postvittana* is an important horticultural pest, mostly of pome fruit, citrus and grapes, and its pest status is a major concern in the USA [5]. In Australasia, the impacts on horticulture are through both direct damage to crops and the market access of exported products. *Epiphyas postvittana* is endemic to mainland Australia, where it is distributed throughout south eastern parts of the landmass, but it has been introduced into Tasmania and countries, such as New Zealand, the USA (Hawaii and California) and northern Europe [1]. The timings of these successful invasions, outside Australia, are thought to vary from the late nineteenth century for New Zealand, early twentieth century for Hawaii, to the late twentieth century for Europe and the early twenty-first century for California. Efforts to determine the origin of the Californian invasion using mitochondrial sequence analysis found evidence for two separate incursions and ruled out a UK or Hawaiian origin for either [6,7].

Due to its pest status and recurrent incursions, there are ongoing efforts to control *E. postvittana* [1]. Insecticides have been an important tool, although resistance, withdrawal of insecticides and the development of alternative measures and Integrated Pest Management programmes have reduced their use. Biopesticides, such as *Bacillus thuringiensis*, Nuclear Polyhedrosis Virus (NPV) and spinosad are effective but not widely used. A number of classical biological control introductions (137) have been made into New Zealand and the Sterile Insect Technique has been considered for controlling the pest [8]. Pheromone-based tactics have been developed for both monitoring and control, using methods such as mating disruption, mass trapping and lure-and-kill. The advent of new gene technologies provides opportunities to develop novel control tactics, such as RNAi [9].

Members of the Lepidoptera (moths and butterflies) have become some of the most serious pests of primary production globally. Their destructive nature is often associated with their ability to complete their life cycle on many different host plants, invade new territories and to be resistant to methods to control them [10,11]. Understanding the biology associated with polyphagy, invasiveness and xenobiotic resistance is now being informed by genomic sequencing of pest species, revealing the genetic basis for these important traits [12]. Indeed, it may well become possible to predict the potential species likely to invade new areas or become pests based on their genetics [13]. More and more genomes of lepidopteran pests are becoming available [14,15,16,17,18], with many being highly polyphagous and invasive. Comparisons of these genomes have identified gene families that are likely to underpin polyphagy, enable invasiveness and confer insecticide resistance [16,18,19]. For example, both polyphagy and invasiveness have been associated with expansion of gene families involved in chemosensation and detoxification [14,16,19,20].

The Tortricidae is one of the largest families of microlepidopteras, which comprise more than 10,000 described species worldwide. The phylogenetic relationships of the Tortricidae, also in relation to the other Lepidoptera, have been studied by Regier and colleagues [21,22]. The family includes a number of important pests of horticulture. Genomic sequences have been deposited in Genbank for eight species, of which six from 2021 are yet to have an accompanying publication. The two genomes with publications are both from pests with relatively narrow host ranges, with codling moth (*Cydia pomonella*) being a pest of apple and related Rosaceae and the smaller tea tortrix (*Adoxophyes honmai*) being a pest of tea. Within the codling moth genome, Wan et al. [17] identified a duplication in an odorant receptor involved in pheromone and host plant recognition, and an association between insecticide resistance and a particular cytochrome P450 (CYP450). From the smaller tea tortrix genome, Uchibori-Asano et al. [23] identified target site resistance and enhanced detoxification by carboxylesterases and CYP450s underlying tebufenozide resistance. To date, however, no highly polyphagous pest within the tortricid family has been genome sequenced.

Here, we describe a genome assembly for *E. postvittana* and compare it with other moth genomes, use it to investigate gene families potentially involved in key pest traits and to inform a population genetic analysis of its invasion patterns.

## 2. Materials and Methods

### 2.1. Genome Sequencing

*Epiphyas postvittana* were from a laboratory colony that has been maintained at Plant & Food Research and its predecessors since 1967. The colony was established from a wild collection from Nelson, New Zealand, with an addition of wild males at generation 128. Larvae were reared on a general all-purpose diet [24]. Pupae were kept at room temperature and separated by sex before eclosion. Adults were provided with sugar and water.

Genomic DNA was extracted from purified nuclei produced using a method based on that of Naim et al. [25] starting from three grams of female pupae (about 50 individuals). We chose only females since in Lepidoptera, females are the usually heterogametic sex allowing a better representation of all chromosomes. Up to 0.2 g of purified nuclei were resuspended in 14 mL of lysis buffer (0.5% SDS, 5 mM EDTA, 150 mM Tris-borate pH 7.4), treated with RNase, DNA purified by chloroform:isoamyl alcohol (24:1) extraction, precipitated with isopropanol and washed with 70% ethanol. The detailed protocol is available at (https://dx.doi.org/10.17504/protocols.io.rncd5aw, accessed on 10 January 2022). DNA quality was assessed at OD 260/280 by nanodrop (Thermo Scientific, Waltham, MA, USA). Mitochondrial DNA contamination was assessed by PCR using primers specific for the mitochondrial *cytochrome oxidase I* gene relative to that of the nuclear gene *Takeout 3*.

Paired-end (PE) and both mate pair (MPE) and Nextera MPE libraries were constructed where the PE insert size varied between 169 bp and 264 bp, and MPE insert size ranged between 2.5 kb and 8.5 kb. Thereafter sequencing was carried out at the Allan Wilson Centre for Genome Sequencing (Palmerston North, New Zealand) and by Macrogen (Seoul, Korea) according to Illumina instructions. Raw paired-end and mate pair reads are available in the SRA archive, with a bioproject ID of PRJNA754242.

### 2.2. Genome Assembly and Assessment

The quality of resulting reads was assessed using FastQC (version 0.9.1). PE data were subject to quality and adapter trimming using the fastq-mcf tool of the ea-util suite (version 1.04.807) [26]. Further quality trimming was carried out using an in-house Perl script to remove reads with Ns and mononucleotides. MPE libraries were quality trimmed using the fastq-mcf tool of the ea-util suite (version 1.04.807), with read length set to 36 bp and PE contamination was removed using Bowtie (version 1.1.1) [27]. Removal of duplicates in both PE and MPE libraries was carried out using an in-house Perl script (available at https://github.com/rosscrowhurst/Perl/blob/fd49a32bd1ef14eca4f085f136afc3b656977547/Fastq/remove_duplicate_read_pairs_orig.pl, accessed on 10 January 2022). MPE data from the Nextera protocol were quality trimmed and duplicates removed using the NextClip tool (version 20140619) [28].

The characteristics of the genome were explored using GenomeScope (version 1) [29], where short-read PE libraries were used as input. Assembly of the genome was carried out using the quality trimmed reads with two assemblers, ALLPATHS-LG (version 50191) [30] and Platanus (version 1.2.4) [31]. The Platanus assembly was further scaffolded with SSPACE (version 2.0) [32] and a round of gap closing using the PE data was performed with GapCloser for SOAPdenovo (version 1.12) [33]. Thereafter both assemblies were checked for plant and microbial contaminants using Kraken (version 0.10.5) [34] where a custom database was made using the invertebrate, plant and microbial RefSeq databases. Subsequently, the NCBI ‘nt’ database was used to further classify the unclassified scaffolds from the first iteration. Based on the Kraken and the NCBI blast classifications, the scaffolds classified as ‘arthropoda’ and ‘unclassified’ were retained and the remainder were discarded as potential contaminants. Finally, Metassembler (version 1.3) [35] was used to merge the free-of-contaminants Platanus and Allpaths assemblies together, with the Platanus assembly set as the primary assembly for the merging.

Completeness of the *E. postvittana* genome was assessed with BUSCO (version 3.0.2) [36], using the ‘arthropoda_odb9′ database, which contains 1066 BUSCO markers and was run in the ‘long’ mode which turns on the Augustus optimization mode for self-training. To further test for completeness of the genome and to investigate the high number of gene models compared with those of other lepidopteran genomes, we mapped the light brown apple moth antennal transcriptomes [37,38] on the genome with Magic-BLAST [39] using default parameters. In addition, genomic reads were mapped back to the genome using Bowtie 2 (v2.3.4.3) [40].

The pattern of coverage and GC% were explored using BlobTools v1.0 [41]. Assembly contigs and scaffolds were blasted against NCBI *nt* database (E-value 1 × 10^−10^).

### 2.3. Genome Annotation

RepeatModeler (v 1.0.8) (http://www.repeatmasker.org; access date 4 March 2022) was used to predict and classify repetitive elements. This tool employs two *de novo* repeat finding programs, RECON and RepeatScout, to identify repeat element boundaries, followed by assigning repeats to the repeat classes based on the sequence feature. The resulting repeat models were searched against the GenBank non-redundant (*nr*) protein database (downloaded on 26 November 2015; E-value < 1 × 10^−5^) using Blastx (v2.2.30) to exclude potential protein-coding genes. The abundances of all predicted repeats were estimated in the genome assembly with RepeatMasker (v4-0-5).

Genome annotation was carried out using AUGUSTUS (version 3.3) [42], where in-house expressed sequence tags (ESTs) [4,37] and repeats were used as hints for the annotation with the BUSCO-trained model generated from the light brown apple moth genome fed in as “--species”. The protein sequences from the Augustus-predicted models were blasted against a protein database containing *Drosophila melanogaster*, *Tribolium castaneum* and the following lepidopteran species: *Amyelois transitella*, *Bicyclus anyana*, *Bombyx mori*, *Chilo supressalis*, *Helicoverpa armigera*, *Papilio machaon*, *Papilio polytes*, *Papilio xuthus*, *Phoebis sennae*, *Pieris rapae*, *Plutella xylostella* and *Spodoptera litura*. Blastp results were fed to OmicBox where mapping and annotation were performed with default parameters.

### 2.4. Comparative Genomics

To identify orthologous groups among *E. postvittana* and other insects we used OrthoFinder [43]. We retrieved protein sequences from the genome of 11 Lepidoptera including one from the superfamily Yponomeutoidea (*P. xylostella*, family: Plutellidae), two from Bombycoidea (*B. mori*, Bombycidae and *Manduca sexta* Sphingidae), three Noctuoidea (*H. armigera*, *S. litura* and *T. ni*, all from the family Noctuidae), three Papilionoidea (*P. xuthus*, *Danaus plexippus*, Papilionidae and two from the family Nymphalidae: *Heliconius melpomene*, *P. rapae*) and a Tortricoidea (*C. pomonella,* Tortricidae). We retrieved the protein data from the genome of a further 10 other insects (two Diptera: *Anopheles gambiae* and *Drosophila melanogaster*; two Coleoptera: *Leptinotarsa decemlineata* and *T. castaneum*; two Hymenoptera: *Nasonia vitripennis* and *Apis mellifera*, two Hemiptera: *Diaphorina citri* and *Bemisia tabaci*; two Blattodea: *Blatella germanica* and *Cryptotermes secundus* and one Collembola, (*Folsomia candida*, Entomobryomorpha). The choice of species was mostly based on the availability of annotated genomes at the time we preformed the analysis, therefore the most recently deposited six Tortricid genomes in Genbank from 2021 were not included. Protein sequences were extracted from genome sequences of each species and cleaned from multiple isoforms. Orthofinder was run, specifying the multiple sequence alignment (-M msa). 

Single-copy orthologues shared by all species were aligned with MAFFT [44] and the resulting alignments were trimmed with trimAl v1.2 [45] to remove poorly aligned regions using the parameter “-automated1”. Single-copy aligned orthologues were merged in a multi-aligned supergene in Geneious. A maximum likelihood tree was obtained with IQ-TREE [46] using the LG+F+R6 substitution model calculated by ProtTest in IQ-TREE. *Folsomia candida* was used as the outgroup.

To identify gene families that significantly changed as a result of a stochastic birth and death process in each branch of the tree we applied the maximum likelihood method implemented in CAFE ver. 5.0 [47]. Using the number of genes per family obtained with OrthoFinder and the IQ-TREE tree as input files, we calculated the average gene expansion and contraction and identified the gene families with significant (*p* < 0.05) size changes (gain and loss of genes) accounting for the species phylogenetic history.

### 2.5. Population Genomics

Ten individuals, both male and female, were sampled from each of ten populations of *E. postvittana.* Two populations were from the North Island of New Zealand, Te Puke (TPU) and Hawke’s Bay (HB); three from the South Island of New Zealand, Motueka (MOT), Awatere (AWA) and Clyde (CLY); four populations from Australia, Wagga Wagga in New South Wales (NWS), Dalkeith in Western Australia (WA), Gumeracha in South Australia (GUM), one in Tasmania (TAS); and one population was from California, USA (CA).

DNA was extracted from each individual and pooled according to population in equal amounts. Each mixture of DNA from 10 moths was cut with Sbf1 for the RAD-tag library to subsample the genome and then sequenced on an Illumina Hiseq. 

Genetic diversity measures for each population, such as nucleotide diversity π, Watterson’s θ and Tajima’s D, were calculated from the pooled samples of each population of *E. postvittana* using the package PoPoolation [48] following the walkthrough available with the software. Briefly, the cleaned reads of each population were mapped to the indexed reference genome (LBAM_v1.0) using bwa [49] with the following parameters (bwa aln -n 0.01 -l 100 -o 1 -d 12 -e 12 -t 8). The resulting sai files were converted to sam files with the function samse of bwa. Ambiguously mapped reads were removed with samtools [50] using the parameters (view -q 20 -bS). The population genetic measures were calculated using a sliding window approach along the genome scaffolds with min-coverage = 10 and min-quality = 20.

Differentiation between populations, expressed as Fst values [51], was calculated for the whole genome as well as for genes and intergenic regions using the package PoPoolation2 [52]. Significance of Fst values weas estimated per gene and intergenic regions by Fisher’s exact tests using PoPoolation2. The parameters used in all Fst calculations were min-count 6, min-coverage 20, max-coverage 2%, pool size 10. Population relationships were also investigated by inferring a maximum likelihood phylogenetic tree with TreeMix [53], grouping SNPs in windows of size 10, since SNPs were very sparse, migration to 3 estimated using the R package OptM [54], and with 100 bootstrap replicates obtained with the R package BITE [55]. The tree was rooted with the Western Australia population of Dalkeith.

## 3. Results

### 3.1. Genome Sequencing and Analyses

**Figure 1:**
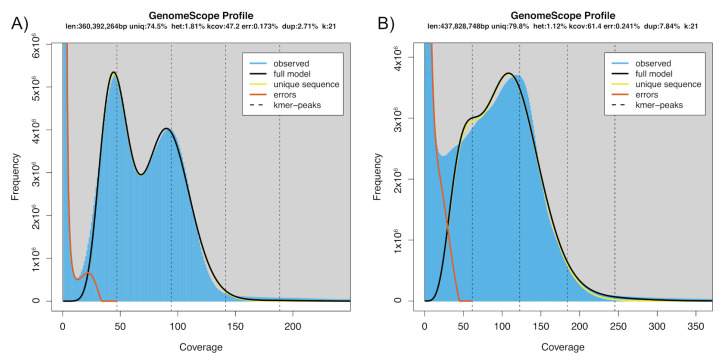
GenomeScope k-mer profile plots for short-read PE libraries illustrating the distribution of 21-mers in (**A**) 169 bp insert size PE data and (**B**) 260 bp insert size PE data.

Genome characteristics using GenomeScope suggested a genome size of 360–437 Mb, with a heterozygosity > 1% (Figure 1). 

Furthermore, the k-mer plots suggested the presence of a high repeat content in the genome, 20–25%. The initial Allpaths assembly size was 539 Mb and over 23,000 scaffolds, with a N50 of 174 kb, while the Platanus assembly was 660 Mb and over 34,000 scaffolds, with a N50 of 248 kb. A contamination check with Kraken, using RefSeq databases, revealed that a high portion of scaffolds were unclassified (~64% in both assemblies) and about 30% of the scaffolds were of arthropod origin, with *Bombyx mori* containing the highest portion (Supplementary Figure S1). Although it appeared that a high portion were categorised as unclassified in relation to assembly size, 493 Mb of the Allpaths and 594 Mb of the Platanus assembly were of arthropod origin, suggesting over 90% of the assembly comprised insects, with a low presence of contaminants. Subsequently, a further 9–11% of the unclassified scaffolds were classified as arthropod using the ‘nt’ database. After contaminant removal, the Allpaths assembly size was 521.6 Mb, with over 20,000 scaffolds, and a N50 of 196 kb, while the Platanus assembly was 638 Mb, with over 32,000 scaffolds, and a N50 of 265 kb. The final merged light brown apple moth genome assembly from Platanus and Allpaths had a total length of over 598 Mb, consisting of over 14,000 scaffolds (Table 1), with a contig N50 of 26.2 kb and a scaffold N50 of 301.17 kb. Assembly length was consistent with the genome size determined by flow cytometry, where the 1C-value was determined to be 0.62 pg, corresponding to an estimated haploid genome size of 606 Mb. Genome completeness level, estimated by BUSCO analysis, was 97.9%, with a duplication rate of 18.3% and 0.6% fragmented, indicating that good representation of the *E. postvittana* genome has been achieved. Furthermore, a high proportion of reads (84–93.5%) were observed to map back to the assembly using Bowtie 2 (v2.3.4.3). 

*Epiphyas postvittana* has 34% of its assembled genome represented as repetitive sequences, with the majority of the known elements made up of longer DNA transposable elements (14.07 Mb) and retrotransposons (LINE 17.83 Mb) (Table 2). The genome assemblies of *E. postvittana* and *P. xylostella* have a similar percentage of the genome represented as repeats, with *H. armigera* having substantially less of the genome represented by repetitive sequences. The LINE family is the most abundant of the modelled element classes in both species. LTRs are more abundant in *P. xylostella*, while SINEs are more abundant in *E. postvittana*. There is a high percentage of unclassified repeats, in both *E. postvittana* and *P. xylostella,* compared with *H. armigera*, indicating that novel repeat classes are yet to be discovered in these species.

### 3.2. Genome Annotation

AUGUSTUS gene prediction resulted in 31,389 de novo predicted genes. This is high compared with those predicted in most other Lepidoptera and more similar to that found in the German cockroach, Blatella germanica (more than 28,000 protein-coding genes) [58], in the parthenogenetic springtail F. candida [59] and in the lepidopteran Heliconius melpomene [60], all with more than 20,000 protein-coding genes. To further investigate this result, we mapped the 23,591 non-overlapping codifying sequences from the antennal transcriptome of E. postvittana male and female [38]. As a result, 90.5% (21,350) of the coding sequences mapped to the genome, with 1406 (6.58%) of them mapping more than once.

The number of gene models with a blast hit against the NCBI protein database was 27,793, of which 27,003 (86%) contained an InterPro domain and 12,276 (38.7%) were assigned a GO term (Supplementary Table S1). No blast hits were recovered for 3596 of the gene models.

### 3.3. Comparative Genomics

**Figure 2:**
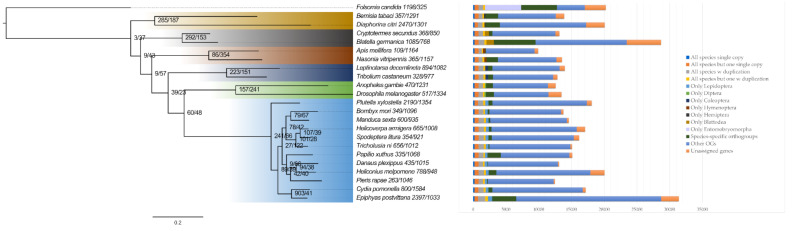
A phylogenetic tree of 22 insect species with Folsomia candida as an outgroup, obtained using the aligned protein sequences of 207 single-copy orthologs present in all 23 species, constructed using the ML method implemented in IQ-TREE. Branch lengths represent substitutions per site. All nodes have a bootstrap support of 100%. Numbers at the nodes and after each species name represent the number of gene families with an increase/decrease in gene number, respectively, estimated by CAFE. On the right, each bar shows the total gene counts per species grouped in the following categories: single-copy genes in all 23 species and in all except one species, gene duplicated in all or except one species, lineage-specific ortholog genes (in Lepidoptera, Diptera, Coleoptera, Hymenoptera, Hemiptera, Blattodea and Entomobryomorpha), species-specific ortholog genes, genes with other ortholog relationships and unassigned genes.

Orthologue analysis with OrthoFinder identified 207 single-copy genes among the 22 insect species and F. candida. The phylogenetic tree from the concatenation of the 207 groups of aligned sequences (80,652 amino acids) is shown in Figure 2. 

Of the 31,389 genes predicted in the *E. postvittana* genome, 28,714 (91.5%) were assigned to 11,438 orthogroups, of which 945 were specific to *E. postvittana*, with 3700 genes predicted to be unique to this species (Figure 2). Over one-third (14,376) of all *E. postvittana* genes share orthologues with the other lepidopteran species examined and 661 of these, in 275 orthologous groups, were unique to members of the Lepidoptera. A full list of the orthologous groups is provided in Supplementary Table S2. Statistical analysis of the evolution of the size of the gene families, using Cafe, given the ML phylogenetic tree and families sizes obtained with Orthofinder, predicted an increase in gene number in *E. postvittana*, across 2397 gene families and a decrease in 1033 from the common tortricid ancestor with *C. pomonella* (Figure 2). A further 903 gene families showed an increase in gene number in the tortricid cluster, which remained constant in *E. postvittana*, with only 41 showing a decrease (Figure 2).

Twenty gene families were significantly expanded in *E. postvittana*, including genes for larval cuticle proteins, genes involved in detoxification, such as cytochrome P450s and glutathione S-transferases (GST), genes involved in transcriptional regulation, such as zinc finger proteins, and genes involved in defence, such as serine protease and macrophage mannose receptors. Among the gene families involved in detoxification, we found almost 300 P450 gene models in 45 orthogroups in the *E. postvittana* genome, with two orthologous groups that showed significant expansion, the CYP 6B and CYP 9E subclasses. Among phase II metabolizing enzymes, more than 50 cytosolic GSTs in 14 orthogroups were detected in the *E. postvittana* genome, with one expanded group of 13 genes, belonging to the sigma class, and more than 100 UDP-glucuronosyltransferase in 16 orthogroups, of which two were expanded in *E. postvittana*.

Only three gene families showed a significant contraction in *E. postvittana*, of which two are associated with transposable elements. The full list is shown in Supplementary Table S2.

### 3.4. Population Genomics

Resequencing of one hundred individuals from ten populations revealed 18,767 SNPs, representing 0.0031% of the 598 Mb assembled genome—the variome. The majority of the SNPs were biallelic, found in genes (57.56%) (Table 3). 

The number of SNPs per population and population genetic parameters are shown in Table 4. 

The lowest number of SNPs, as well as the lowest nucleotide diversity (π), were found in Te Puke (3669 SNPs and 7.12 × 10^−4^, respectively), whereas the highest number of SNPs were found in California (7934), and the highest π in Gumeracha (1.24 × 10^−3^). The lowest population mutation rate (θ) was from Clyde (8.14 × 10^−4^), whereas the highest was in Gumeracha (1.48 × 10^−3^). Tajima’s D values were only slightly negative for all populations. There were no significant differences in any population genetic parameters among countries (Krustall–Wallis test: π: χ^2^ = 4.55, df = 2, *p* > 0.05, θ: χ^2^ = 3, df = 2, *p* > 0.05, D: χ^2^ = 1.59 df = 2, *p* > 0.05).

Average Fst among all populations was 0.146. Genetic differentiation was similar among populations, with the lowest Fst value being between the two North Island populations of New Zealand, Hawke’s Bay and Te Puke (0.086) (Table 5). 

**Figure 3:**
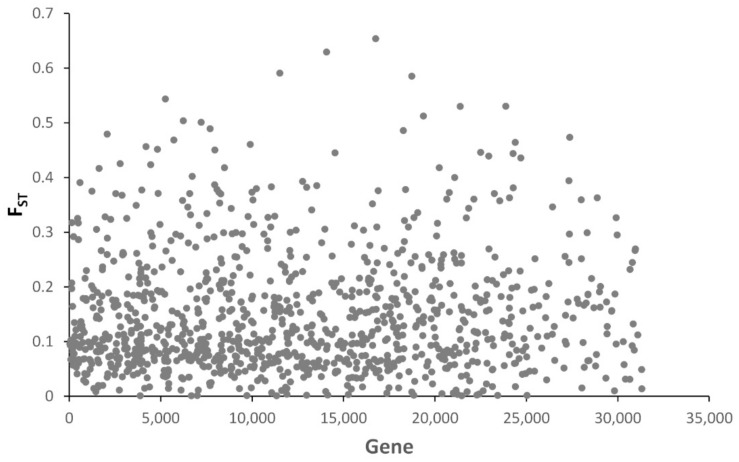
Distribution of average Fst values among populations of *Epiphyas postvittana* calculated from the SNPs within gene models in the genome.

Of the 31,389 predicted genes from the assembled *E. postvittana* genome, 1025 (4.5%) contained SNPs and were informative for estimating the differentiation among populations (Figure 3). However, population differentiation was similar when calculated using SNPs within genes versus within intergenic regions (0.147 and 0.144, respectively) (Supplementary Table S3), and their pairwise population values were highly correlated (r^2^ = 0.914). Of these 1025 genes, 51 were in the top 5% of the Fst distribution (Fst > 0.37) and 50 of these genes were among the annotated genes (Supplementary Table S4). 

No GO enrichment was detected among the outlier genes. Genetic differentiation among populations was correlated with their geographic distances, whether considering all SNPs (Corr. = 0.970321, *p* = 0.05) or either genic or intergenic regions (Corr. = 0.966436, *p* = 0.05; Corr. = 0.974291, *p* = 0.05, respectively).

**Figure 4:**
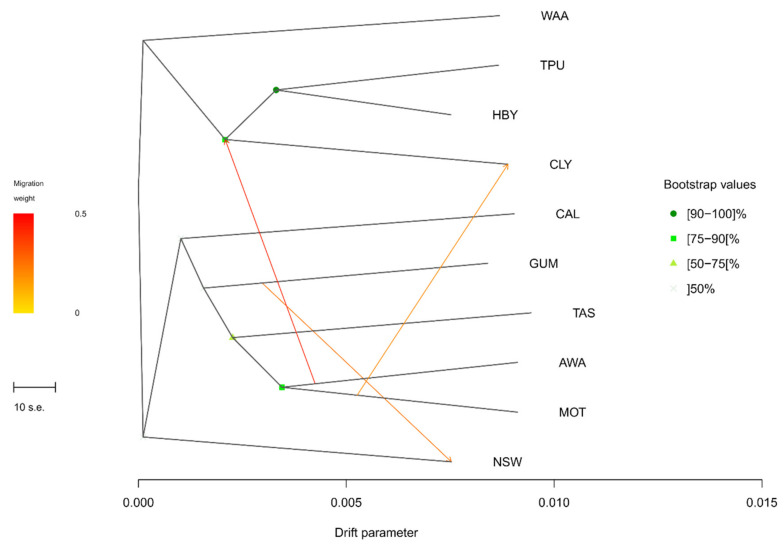
Historical relationships and inferred migration events (indicated by the coloured arrows) among Epiphyas postvittana populations obtained with TreeMix.

In a phylogenetic analysis (Figure 4), using TreeMix, the New Zealand populations formed two clusters, with the two most northern populations from the South Island (MOT and AWA), sister to the Tasmanian population. The most southern population from the South Island (CLY) was sister to the two North Island populations (TPU and HBY). The Californian population, clustered together with the eastern Australian populations (GUM, TAS and NSW) and two of the South Island populations of New Zealand (AWA and MOT). Migration events were predicted between the South Island populations of New Zealand and between Wagga Wagga in New South Wales and Gumeracha in South Australia.

## 4. Discussion

In this paper we present the first genomic resources for the light brown apple moth, *E. postvittana*, an invasive and polyphagous lepidopteran pest of horticultural systems. We further used this genomic resource to investigate the expansion of gene families putatively linked to the evolution of polyphagy and the population genomics of this invasive species. 

The predicted size from the genome assembly of 598 Mbp is similar to the genome size determined by flow cytometry of 606 Mbp, and is within the range of those of other Lepidoptera sequenced to date. The size of the *E. postvittana* genome does not seem due to an expansion of repetitive elements, which make up 33.8% of the genome (Table 2), a proportion lower than in *B. mori* and *P. xylostella,* which both have smaller genomes than *E. postvittana*. Additionally, the percentage of different types of repeat elements was quite similar to that found in other lepidopteran genomes, although there were a high number of unclassified repeats (26%), as was reported for the *P. xyolstella* genome (28%) [15]. The K-mer profiles (Figure 1) suggested high heterozygosity and high repetitiveness, probably due to the high number of individuals used to construct the sequencing libraries, suggesting difficulty in assembly, although this genome assembly is only based on Illumina reads. However, high and similar levels of heterozygosity have been found from the genome sequencing of a single individual of the inbred clonal honey bee, *Apis mellifera capensis* [61], as well as other insects we have sequenced (unpublished data). Nevertheless, additional resolution of the *E. postvittana* genome would probably benefit from the newer long-read technologies, as well as using high molecular weight genomic DNA extracted from a single insect.

In comparison with genome size, the gene number is reasonably high compared with those of other lepidopteran genomes (Table 1), which may well be related to genome assembly only being based on Illumina reads. However, the number of genes that are expressed in the antennae of both sexes [38] was also higher than in the gene models of other lepidopteran genomes, and most of these CDS (21,350; 90.5%) mapped to our genome assembly. This evidence may suggest that the *E. postvittana* genome could contain a higher number of genes than those of other sequenced members of the Lepidoptera.

The evolution of highly polyphagous species has been linked to the expansion of gene families involved in detoxification pathways, from active metabolism of toxic plant compounds to enhanced excretion [14,16,19,20]. These same gene families have also been implicated in the development of resistance to pesticides in many insect species, suggesting a tight link between polyphagy and ability to develop resistance to xenobiotics [62,63]. We found several gene families that significantly changed in copy number, in the lineage leading to *E. postvittana*, with expansions in gene families involved in detoxification, defence and cuticle structure. Among the families involved in detoxification, we found expansion in phase I metabolism genes, including two CYP450 families, and phase II metabolism genes, including members of the sigma class of GSTs and two UDP-glucuronosyltransferase (UGT) families. While it is difficult to determine whether these expansions are biologically real or artefacts of the assembly process, given the source DNA was from multiple individuals, the observed expansions are in gene families known to be expanded in other Lepidoptera [18,19,56,64]. Furthermore, the antennal transcripts that mapped multiple times on the *E. postivittana* assembled genome did not involve the GST family, and in only one case, the P450 6B and UDP orthogroups, out of 38 and 23 changes in gene copy number in *E. postvittana*, respectively. 

For CYP450s, there is good evidence that new copies appear when herbivores colonize new host plants [65,66,67], supporting the hypothesis that an arms race between herbivorous insects and their host plants drives P450 diversification. Of the two expanded groups of CYP450 in *E. postvittana*, members of the 6B subfamily are able to metabolize linear furanocoumarin (xanthotoxin and bergapten) in the lepidopterans *Papilio polyxenes* and *P. glaucus* [68], whereas members of the A and E subfamilies are highly upregulated in insecticide-resistant strains of *Aedes aegypti* and *Culex pipiens* [69,70].

GSTs have an important physiological role in lipid peroxidation by eliminating by-products of oxidative stress, as well as roles in insecticide resistance and in the breakdown of plant protective compounds [71,72]. Sigma GSTs are ubiquitous among metazoans and are one of the most abundant and conserved GST classes in insects [73]. The expansion of the sigma class in the light brown apple moth is a new report amongst the Lepidoptera, where typically expansions of the epsilon and delta classes have been observed [20]. Both the epsilon and delta classes have been implicated in detoxification and resistance to insecticides [19,71]. These two classes contained 20 and 7 gene copies, respectively, in *E. postvittana*. 

UGTs are membrane-bound phase II metabolizing enzymes that, in insects, conjugate glucose to various endogenous and exogenous substrates, playing an important role in the detoxification of plant allelochemicals by promoting their excretion [74,75]. Most UDPs are highly expressed at larval stages, indicating their importance in the protection against plant secondary metabolites [76]. However, UGTs have also been implicated in insecticide resistance because of their activity on xenobiotics [77,78] and, in addition, they play roles in several other processes, including cuticle formation and olfaction [74]. The two UDP families that are expanded in *E. postvittana*, UDP33 and 40, are the two largest UDP families in both *B. mori* and *P. xylostella*, with the expression of most of their members being affected by insecticide treatments [78].

Mechanisms to minimize the effects of ingestion of plant proteinase inhibitors in insects often involve the upregulation of serine peptidases of the chymotrypsin and trypsin family [79]. In *Spodopera frugiperda*, chronic exposure to proteinase inhibitors results in the upregulation of 16 chymotrypsin and trypsin genes. The responsive trypsin genes belong to a separate clade from unresponsive genes, supporting a possible mechanism of resistance involving gene number expansion [80]. This same gene family has also been implicated in insecticide resistance [81].

In other highly polyphagous pests, such as *H. armigera* and *S. litura*, an expansion of gustatory receptors has been observed, which is thought to allow the detection of the many host defence response compounds [19]. This was not seen in *E. postvittana*, however, we did notice that manual efforts to annotate this gene family resulted in higher estimates in the number of gustatory receptor genes in *E. postvittana* (unpublished data), in line with other polyphagous Lepidoptera [19]. The annotation of odorant receptors genes and other genes involved in odorant reception has been covered separately [37].

The light brown apple moth is a global pest. From Australia, it invaded New Zealand in the late 19th century [82], and soon after, colonized Hawaii, between 1900 and 1925 [83], then England, where it arrived in 1936 [84]. More recently it has invaded Ireland (1968), Sweden and the Netherlands, and in 2006, California [1,85,86]. The resequencing of pooled samples from ten *E. postvittana* populations found more than 18,000 SNPs, of which more than 50% were within gene models. Although the number of pooled individuals was low for a very accurate estimation of variant frequency, genomic population results were similar to those obtained from a survey of mtDNA variation [7]. All populations were similarly diverse, including those in Tasmania, which showed higher pairwise Fst than observed for mtDNA [7]. Similar to the mtDNA results, California showed the same degree of diversity as Australia and New Zealand, despite its more recent origin. On the basis of the mtDNA haplotypes of the 2006/2007 samples, Tooman et al. [7] suggested two probable invasions in California. The high genetic diversity in the 2007 samples also indicated either a source that was highly diverse genetically, or multiple sources, for which both Australia and New Zealand fit the profile. Our genomic data identify Australia as a probable source of the California invasion, as suggested by population relationships on the ML tree, in which California clusters with the population of Wagga Wagga and Gumeracha in South Eastern Australia (Figure 3). New Zealand was likely invaded twice, as shown by the two distinct clusters in Figure 3, and with signs of admixture of populations from the South Island to the North Island. Signs of admixture were also evident between the two closest populations in Australia, Wagga Wagga and Gumeracha. Nevertheless, this result should be taken with caution, since both the sample size within populations and the number of sampled populations are low.

In the USA, the light brown apple moth is classified as a Class A pest. Recent models predict its potential invasive range reaching most of the coastal areas of the globe, USA and central Mexico, South America in Chile, Perù and Argentina, most of western Europe, and all the Mediterranean basin, the south of the African continent and Southeast Asia, including inland areas [87]. The highly polyphagous nature of *E. postvittana*, its high rates of genetic diversity, and predictions of its potential range, including many horticulturally important growing regions, raises the degree of concern about this species. As such, the genomic resource presented here should be useful in developing new management tools for this pest and for understanding the biological basis for this and other species’ ability to invade new regions.

## Figures and Tables

**Table 1:** Summary statistics of the assembly, features and quality assessment of the *Epiphyas postvittana* genome in comparison with those of other lepidopteran species.

Species	*Epiphyas postvittana*	*Cydia pomonella* [17]	*Trichoplusia ni* [14]	*Helicoverpa armigera*	*Plutella xylostella* [56]	*Spodoptera litura* [19]	*Bombyx mori* [57]
Genome assembly:							
Assembly size (Mb)	598.1	772.9	333.0	337.0	394.1	438.3	431.7
Number of scaffolds	14,077	1717	1916	997	1819	3597	43,622
Max scaffold length (Mb)	3.70	34.60	8.92	6.15	3.94	4.40	16.12
N50 scaffold size (kb)	301.17	8915.45	4648.10	1000.40	737.18	915.40	3717.00
Number of contigs	46,180	2221	7885	24,228	14,357	13,636	88,842
N50 contig length (kb)	26.2	862.5	140.0	18.3	49.4	68.4	15.5
Genomic_features:							
Protein coding gene	31,389	17,184	14,384	17,086	18,071	15,317	14,623
Repeats (%)	33.8	42.9	16.7	14.6	34.0	31.8	43.6
GC (%)	38.3	37.4	35.5	36.1	38.4	36.6	38.8
Mean CDS length (bp)	1204.0	1461.0	1512.3	1330.9	1385.0	1565.2	1212.7
Mean exon length (bp)	230.0	256.9	316.7	309.2	214.0	235.8	222.2
Mean intron length (bp)	1254.0	1205.3	1603.3	1379.4	1224.0	1938.4	1084.1
Mean number of exons per gene	5.20	5.68	7.04	6.20	6.47	6.64	5.45
Quality_assessment: BUSCO % present * (complete)							
Genome	98.5 (97.9)	98.5 (97.8)	98.8 (98.4)	98.5 (97.1)	92.7 (89.9)	99.0 (98.4)	94.5 (92.1)
Protein (OGS)	95.7 (92.0)	93.8 (90.6)	97.8 (97.3)	98.4 (96.0)	80.1 (75.0)	99.0 (97.9)	96.3 (91.5)

* Present = complete + fragmented.

**Table 2:** Repeat classes in the *Epiphyas postvittana* genome compared with those of other Lepidoptera.

Species	*Epiphyas postvittana*		*Cydia pomonella*		*Trichoplusia ni*		*Helicoverpa armigera*		*Plutella xylostella*		*Spodoptera litura*		*Bombyx mori*	
Repeat Types	Length (Mb)	P%	Length (Mb)	P%	Length (Mb)	P%	Length (Mb)	P%	Length (Mb)	P%	Length (Mb)	P%	Length (Mb)	P%
DNA elements	14.07	2.35	26.95	3.49	21.92	6.58	0.22	0.45	7.50	1.90	8.4	1.96	69.07	10.44
LINE	17.83	2.98	68.51	8.86	6.66	2.00	0.13	0.27	20.62	5.23	46.27	10.81	101.25	15.31
LTR	4.59	0.77	11.39	1.47	1.51	0.45	0.06	0.12	9.86	2.5	2.41	0.56	4.55	0.69
SINE	6.12	1.02	21.03	2.72	5.47	1.64	-	-	2.01	0.51	8.51	1.99	8.62	1.30
Simple repeat	5.11	0.85	5.08	0.66	3.19	1.92	-	-	-	-	4.52	1.06	6.26	0.95
Other	0.51	0.09	1.81	1.39	3.19	1.92	-	-	0.000057	0	0.67	0.16	0.93	0.14
Unclassified	153.74	25.71	-	-	16.79	5.04	0.08	0.16	111.25	28.23	73.89	17.26	54.55	8.25
Total	201.97	33.77	341.54	42.87	55.54	16.68	49.00	14.60	151.24	38.37	136.17	31.8	245.23	37.33

**Table 3:** Number of SNP types, percentage and distribution in genic and intergenic regions of the Epiphyas postvittana genome.

SNP Type	Genic	Intergenic	Total
Biallelic	10,803 (57.56)	5986 (31.9)	16,789 (89.46)
Triallelic	1261 (6.72)	585 (3.12)	1846 (9.84)
Tetraallelic	96 (0.51)	36 (0.19)	132 (0.70)
Total	12,160 (64.79)	6607 (35.21)	18,767 (100.0)

Among brackets % calculated on the total number of SNPs.

**Table 4:** Epiphyas postvittana population genetic parameters per population.

Country	Population	N	pi	Theta	D
AUS	Gumeracha (GUM)	7052	0.0012	0.0015	−0.0325
	Wagga Wagga (NSW)	5998	0.0012	0.0014	−0.0423
	Tasmania (TAS)	5006	0.0009	0.0010	−0.0291
	Dalkeith (WAA)	5890	0.0009	0.0012	−0.0352
USA	California (CA)	7934	0.0009	0.0011	−0.0282
NZ	Awatere (AWA)	4325	0.0009	0.0010	−0.0339
	Clyde (CLY)	4005	0.0007	0.0008	−0.0259
	Hawke’s Bay (HB)	4523	0.0009	0.0011	−0.0373
	Motueka (MOT)	5625	0.0009	0.0011	−0.0299
	Te Puke (TPU)	3669	0.0008	0.0009	−0.0422

**Table 5:** Average FST across the whole genome of *Epiphyas postvittana* (calculated as average of all gene and intergenic FSTs). For population abbreviation, see Table 4.

	GUM	NSW	TAS	WAA	CA	AWA	CLY	HB	MOT	TPU
GUM	-	0.10611	0.1199	0.14067	0.12127	0.10816	0.15075	0.11949	0.11523	0.14398
NSW		-	0.14548	0.1359	0.13567	0.12461	0.16092	0.12251	0.13378	0.14405
TAS			-	0.1885	0.16099	0.12862	0.18155	0.15695	0.13225	0.19199
WAA				-	0.17158	0.16146	0.1973	0.1635	0.1703	0.18416
CA					-	0.14844	0.19214	0.1566	0.15068	0.18032
AWA						-	0.14505	0.11311	0.09855	0.14918
CLY							-	0.12674	0.14836	0.15415
HB								-	0.13135	0.08647
MOT									-	0.16317
TPU										-

## Data Availability

All raw sequencing data have been deposited in the SRAS archive, with bioproject ID PRJNA754242 in the NCBI database.

## Supplementary Materials

The following are available online at https://www.mdpi.com/article/10.3390/insects13030264/s1, Table S1: Blast description, GO and InterPro annotation of gene models obtained from Augustus gene prediction; Table S2: List of all orthologous groups recovered by OrthoFinder with a representative gene model, where present, of *Epiphyas postvittana* and its blast description, GO and InterPro annotation. For each orthologous group the number of gene changes in the gene tree are indicated; Table S3: Average Fst calculated on genic SNPs (above the diagonal) and on the intergenic SNPs (below the diagonal); Table S4: List of 50 genes in the highest 5% of the Fst distribution and their relative blast hit, description and annotation; Figure S1: Krona plot illustrating the taxonomic classification of the scaffolds using the RefSeq sequences as the reference database.
