# Supplementary material for: The Genomics and Population Genomics of the Light Brown Apple Moth, Epiphyas postvittana, an Invasive Tortricid Pest of Horticulture
Source: Insects. 2022 Mar 7;13(3):264. doi: 10.3390/insects13030264 (PMC8951345; doi:10.3390/insects13030264)
Supplement: Supplementary file 1 [file insects-13-00264-s001.zip › insects-1580622-supplementary/Figure S1.pdf]

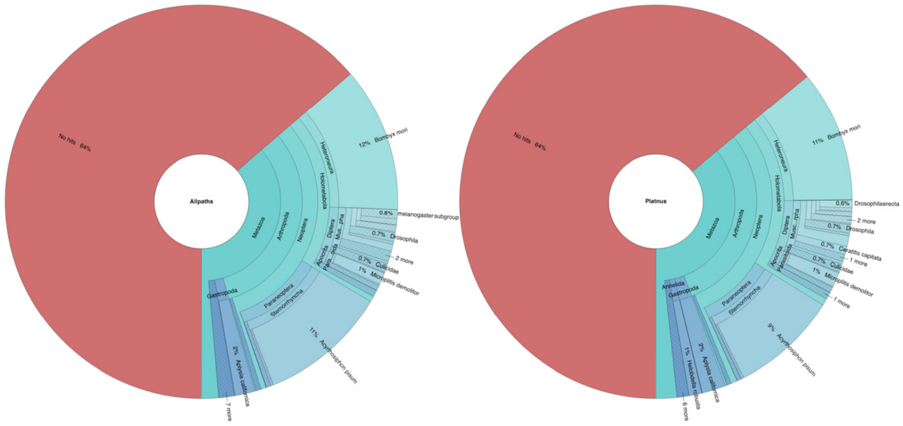

Figure S1. Krona plot illustrating the taxonomic classification of the scaffolds using the RefSeq sequences as the reference database.
